# Greenhouse Gas Emissions Associated with Nile Tilapia (*Oreochromis niloticus*) Pond Fertilization in Western Kenya

**DOI:** 10.1155/2023/1712985

**Published:** 2023-05-26

**Authors:** S. A. Odinga, A. Sifuna, H. Lungayia, G. Wanyama

**Affiliations:** ^1^Fisheries Department, Kakamega County, P.O. Box 586-50100, Kakamega, Kenya; ^2^Masinde Muliro University of Science and Technology, Department of Biological Sciences, P.O. Box 190-50100, Kakamega, Kenya; ^3^International Livestock and Research Institute, P.O. Box 30709, Nairobi, Kenya

## Abstract

In the recent past, fish farming has gained great prominence in Kenya as the country straggles to meet food security. Nile tilapia (*Oreochromis niloticus* L.) farming has attracted the most demand, with the use of manure to enhance primary productivity in fish ponds being encouraged as a form of increasing productivity and returns on investment. The objective of this study was to understand the role of Nile tilapia farming in greenhouse emissions (GHGEs) in the region. Generally, there is paucity of such information originating from sub-Saharan Africa. Here, we report the levels of methane (CH_4_), carbon dioxide (CO_2_), and nitrous oxide (N_2_O) emissions from Nile tilapia fish ponds fertilized with organic and inorganic fertilizers. We also try to establish if there exists any relationship between GHGEs and physicochemical parameters (PCPs). The methane fluxes ranged from 0.001 to 0.043°mg·m^−2^h^−1^ in UF ponds, 0.005 to 0.068°mg·m^−2^h^−1^ in IF ponds, and 0.001 to 0.375°mg·m^−2^h^−1^ in OF ponds. The findings show that the fluxes were significantly different (*P* < 0.05). Mean fluxes of CO_2_ did not show significant difference among the treatments (*P* > 0.05), ranging from −0.180 to 1.40°mg·m^−2^h^−1^ in UF ponds, −0.020 to 1.101°mg·m^−2^h^−1^ in IF ponds, and −0.049 to 1.746°mg m^−2^h^−1^ in OF ponds. N_2_O mean fluxes were not significantly different (*P* > 0.05), ranging from −0.628 to 0.326°*µ*gm^−2^h^−1^ in UF ponds, −0.049 to 0.187°*µ*gm^−2^h^−1^ in IF ponds, and −0.022 to 1.384°*µ*gm^−2^h^−1^ in OF ponds. UF had a mean flux of −0.003 ± 0.175°*µ*gm^−2^h^−1^, IF had a mean flux of 0.032 ± 0.056°*µ*gm^−2^h^−1^ and OF had a mean flux of 0.093 ± 0.324°*µ*gm^−2^h^−1^. There was significant difference in the carbon to nitrogen (CN) ratio among the fertilization treatments (*P* < 0.05), whereas temperature, pH, dissolved oxygen, and conductivity showed no significant difference among the fertilization treatments (*P* > 0.05). The study observed that fertilization of Nile tilapia ponds significantly increases the release of CH_4_ emission and the CN ratio. Temperature, conductivity, and CN positively correlated with CH_4,_ CO_2_, and N_2_O emissions. Dissolved oxygen showed a negative correlation with CH_4_ and CO_2_ emissions while negatively correlated with N_2_O emissions. The study identified the use of OF as a potential form of fish farming that promotes the emission of GHGEs and calls for adoption of sustainable technologies for the management of organic and inorganic fertilizers before their use in pond fertilization.

## 1. Introduction

Global warming has emerged as a major global challenge, and all governments in the world have been called to action [1]. One of the actions, governments are to undertake, is to cut greenhouse gas (GHG) emissions. To achieve this, governments need to understand which sectors are responsible for how much GHG emissions. In this study, we aimed at estimating the amount of methane (CH_4_), carbon dioxide (CO_2_), and nitrous oxide (N_2_O) gases emitted into the atmosphere from Nile tilapia farming. Generally, there is little information on how fish farming influences GHG emissions in the region. In the last 15 years, fish farming in Kenya has increased 10-fold, increasing from 271 ha to over 2500 ha [2], therefore calling for a need to a better understanding of the types and quantity of the emission. The agricultural sector has been estimated to be the largest source of GHG emissions of all sectors in Kenya [3], and about 40% of total national emissions in 2015 were from this sector alone [4].

*Oreochromis niloticus* remains the most widely cultured fish species in the world because of its high consumer acceptance, high commercial value, and flexible eating habits that allow for the use of high levels of plant protein, making it easier to adapt easily to most fish culture systems with increased intensification [5]. However, longstanding hurdles to enhancing Nile tilapia production in the supply of quality fish feeds remain a challenge [6]. Supplementary feeds are the most expensive input in intensive and semi-intensive cultures [7]. Combining fertilization and supplemental feeding has been shown to reduce production costs [8]. According to [9], fish pond manuring is used in fish farming for the intensification of fish production by balancing the ratio between carbon and other nutrients. Odinga et al. [10] demonstrated that fish pond fertilization, whether with organic or inorganic fertilizer improved the growth of Nile tilapia in smallholder earthen ponds. At the same time, it has been shown that GHG emissions tend to increase as an aquaculture system moves from being extensive (untreated or partially fertilized) to semi-intensive (fertilized and/or partially fed) and intensive (completely fed and fertilized) [11]. These fertilizers are introduced in the fish pond water, which forms the immediate environment for fish and is very important as it forms the basis of fish metabolism [12].

The rapid expansion in aquaculture has received negative reputation for its associated environmental impacts [13]. There have been calls for sustainable intensification (SI) in order to produce more using less in a bid to increase productivity and environmental impacts arising from aquaculture [14]. According to [15], aquaculture remains one of the best innovative agricultural options for achieving food security under changing climate, but with the effects of climate change in aquaculture becoming more prevalent, research efforts are being directed towards developing and validating climate smart aquaculture (CSA) technologies, innovations, and management practices (TIMPs) for sustainable fish production [16]. Therefore, in order for the region to mitigate GHG emissions, there is a need to understand how existing systems perform. This study therefore identified and quantified sources of GHG emission from aquaculture systems in Western Kenya.

## 2. Materials and Methods

### 2.1. Study Area

**Figure 1:**
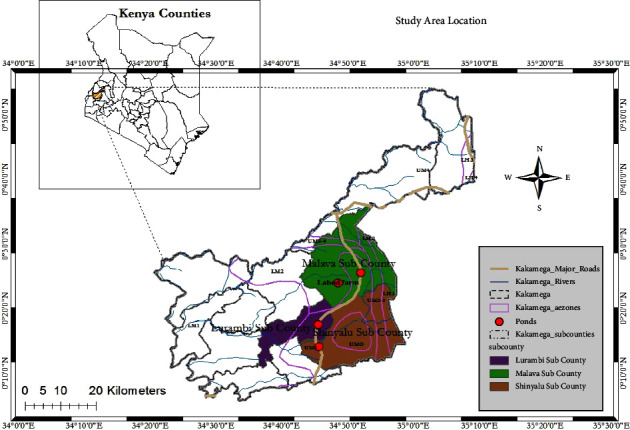
Map showing the study sites.

The study was carried out in Kakamega County, as shown in Figure 1 (0.2827°N, 34.7519°E), found in Western Kenya. It covers an area of 1,395 km^2^ with a population of 1,868,000 [17]. Kakamega is mainly tropical, with variations due to altitude with an average elevation of 1,523 meters [18]. It experiences heavy rainfall all year round, with two seasons, namely, the long rains (April to July) and short rains (August to November). Rainfall ranges from 156 to 663 mm/month with a temperature range of 9.95–26.0°C [18]. The coldest month is July, with an average of 9.95–12.0°C, whereas the hottest season is experienced between December and February, with a temperature range from 24.5–26.0°C [18].

Kakamega County is well endowed with a vast water resource that can be harnessed for fish farming. In 2018, Kakamega County had 7,939 fish farmers operating 8,540 fish ponds covering an area of 2,260,945 m^2^ [19]. In the same year, 1,730,000 fingerlings of Nile tilapia and catfish fingerlings valued at Kshs. 13 million were stocked in the county. Fish weighing approximately 1,600 t, valued at about KES. 500 million, were harvested and sold in the same year [19]. The large numbers of smallholder fish farmers that fertilize their fish ponds using animal manure cannot be ignored, and this calls for the need to assess their contribution to the environment, apart from fish production.

### 2.2. Study Design

Our study focused on the emissions during pond post application. An experimental study involving three fish farms in Kakamega County, each with three ponds measuring 300 m^2^ and a depth of 1 m, was adopted. The ponds were purposively selected in a randomized complete block design (RCBD). The choice of fertilization method in this study was based on a survey conducted among the fish farmers of Kakamega County, which revealed that 87.6% of fish farmers fertilized their ponds, while 12.4% did not fertilize their ponds. Among the farmers fertilizing their ponds, 64% used animal manures (41% chicken manure, 49% cow dung, and 11% other manures), while 36% used inorganic fertilizers. On each of the farms, the three ponds consisted of an unfertilized pond (UF), inorganic fertilizer fertilized pond (IF), and organic manure fertilized pond (OF). Stocking and feeding of the fish were done from June 2021 to December 2021. Fish ponds used in this study were earthen Nile tilapia ponds, which were well-constructed and retained water throughout the cycle, with initial pond filling at the beginning of the cycle, followed by weekly water top-up to compensate for evaporation.

### 2.3. Sampling

#### 2.3.1. Sampling of Manure and Feeds

The manure and feeds were aseptically sampled into 250 ml autoclaved propylene bottles by picking from 5 different points and mixing for uniformity. 100 g of each sample manure and compost were transferred into the bottles for analysis of the carbon to nitrogen ratio. The samples were transported to the laboratory in a cooler box packed with ice. This was done in three batches during the study.

#### 2.3.2. Sampling of Water

The water samples were collected aseptically using presterilized 250 ml glass bottles, which were submerged 15 cm to 20 cm below the water with the mouth facing upwards. The glass bottles were thoroughly washed with detergent, rinsed with tap water and soaked for 6 hours in 20% (v/v) of hydrochloric acid, and rinsed with reagent water to remove all traces of organic materials which could cause CN contamination. Samples were taken by filling the bottles to the top to exclude air. Bottles with water samples were labeled accordingly. Water samples were placed in a cool box packed with ice and then transported to the laboratory for total carbon and total nitrogen analysis on a monthly basis.

#### 2.3.3. Sampling of Gases

**Figure 2:**
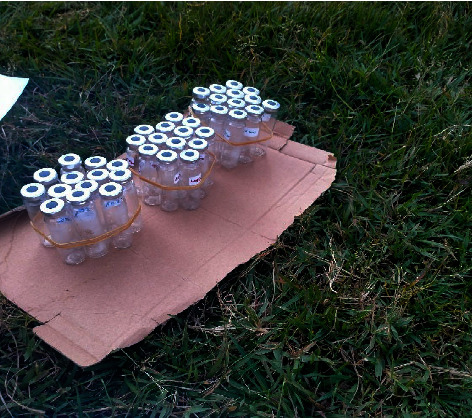
Glass vials fitted with a rubber seal for taking gases to the laboratory for analysis.

Every time gases were sampled, supporting measurements of chamber temperature and ambient temperature were performed using Wertheim EN 13485 thermometer and atmospheric pressure using a phone-installed barometer. Static chambers were used to capture concentrations of CO_2_, CH_4_, and N_2_O. Concentrations were captured by deploying 3 chambers per pond on a monthly basis according to [20]. The concentrations were captured at each of the three points at intervals 10 minutes for 30 minutes using syringes and pooled in 60 ml previously evacuated glass vials sealed with butyl rubber septum (Figure 2), for transportation to the lab for analysis [21]. The pooling was done to form a composite air thereby overcoming spatial heterogeneity as recommended in [22]. Four gas samples were taken, sequentially from time zero to 30 min (at intervals of 10 minutes) to be able to calculate the flux rate [23]. Sampling was done between 10 am and 12 am when the temperatures reflected the daily averages [21].

**Figure 3:**
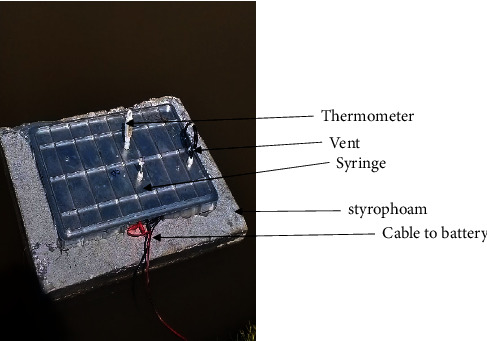
Picture of a locally fabricated chamber used to capture gas concentrations in fish ponds.

The chambers were locally fabricated according to recommendations in [24]; typically, chambers cover an area of 0.1–1 M^2^, should have a vent tube to maintain pressure equilibrium, using Styrofoam material (silver in color), and should be reflective to reduce solar radiation and able to float. Our chambers were rectangular, measuring 0.56 M length, 0.38 M width, and 0.1 M height (Figure 3).

### 2.4. Pond Preparation, Fertilization, Stocking, and Fish Feeding

#### 2.4.1. Pond Preparation

The nine 300 m^−2^ earthen fish ponds were drained of their contents (water, fish, and plants). They were then limed using quick lime (CaO) at a rate of 200 g·m^−2^ (one off) and left for 7 days before fertilization.

#### 2.4.2. Fertilization

The ponds were filled with water as the fertilization regime was performed, i.e., UF being unfertilized, IF fertilized using di-ammonium phosphate, applied weekly at 2 g·m^−2^/week whose NPK ratio was 18 : 46 : 0 (soaked in water and broadcasted in the pond water), and OF, whose organic manures used were chicken manure (carbon to nitrogen ratio was 13.76) and cow dung manure (carbon to nitrogen ratio was 13.83), applied at the rate of 20 g·m^−2^/week. The ponds were left for 7 more days before stocking with fingerlings, after which fertilization followed weekly.

#### 2.4.3. Stocking of Fingerlings

**Figure 4:**
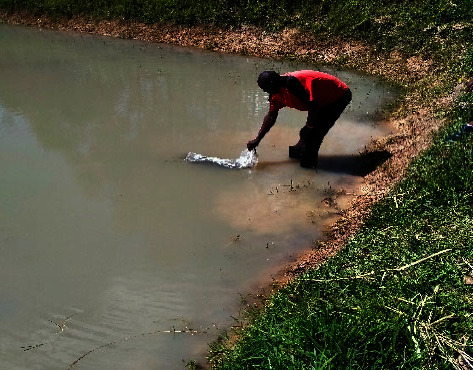
Picture showing Nile tilapia fingerlings being stocked in one of the study ponds.

All the ponds were stocked with 1,000 all-male Nile tilapia fingerlings (*Oreochromis niloticus* L.) from the same hatchery, with an average weight of 0.5 g and an average length of 1.9 cm (3 fish per m^−2^ plus 100 mortality allowance). The fingerlings were stocked at 6 weeks of age after hatching (Figure 4). The fingerlings were sourced from Labed Cash Hatchery Ltd.

#### 2.4.4. Fish Feeding

The fish were fed at 5% average body weight in the first two months, 3% average body weight in the next two months, and 2% average body weight in the last three months with fish feed with the following proximate compositions listed in Table 1.

The carbon to nitrogen ratio of the feed that was fed to fish was 6.82.

### 2.5. Physicochemical Parameters

Water quality parameters, including temperature, dissolved oxygen, pH, and conductivity, were measured *in situ* using a Hydrolab MSIP-REM-HAH-QUANTA (USA) at three points of each pond (inlet, middle, and outlet). This was done on a monthly basis in all the replicates between 9 am and 11 am.

#### 2.5.1. Carbon to Nitrogen Ratio in Pond Water Samples

Total dissolved C and N in water samples were determined by TOC/TN-analysis on a TOC-L CPN model Shimadzu analyzer. TDN was quantified by high temperature catalytic oxidation (HTCO) at 680°C, and a platinum catalyst was used to complete the oxidative conversion of all forms of C to CO_2_ and all forms of N to NO and NO_2_. NO and NO_2_ then reacted with O_3_, producing an excited state of NO_2_ (NO_2_^*∗*^). Upon returning to the ground state, light energy is emitted which is quantified by chemiluminescence detection. The content of total organic carbon (TOC) was then determined by the difference method (TOC = TC−TIC) as well as the addition method (TOC = POC + NPOC), where TOC was measured as nonpurgable carbon (NPOC) where after in-syringe acid addition of acid, the samples were purged with synthetic air to release inorganic carbon (TIC). The 100 ml water samples for total C and N analysis were filtered on a GF/F filter (0.45 *μ*m) using a vacuum pump (pressure 200 mm Hg). GF/F filter paper was first heated in an oven at the temperature 105°C for 2 hours to attain constant weight. The contents were then weighed on a scale (Mettler Toledo-XP205) and the weight was given as mgl^−1^ of total carbon or total nitrogen.

The carbon to nitrogen ratio was calculated by the following equation:

(1) $$\begin{array}{c} CN=\frac{TC}{TN}\text{.} \end{array}$$

#### 2.5.2. Carbon to Nitrogen Ratio in Manure and Feed Samples

Manure and compost samples were oven dried at 60°C in a forced-air oven overnight. From the dried samples, 0.2 g aliquots were weighed into steel crucibles and ignited on an Elementar Vario Max Cube model CN analyzer in the abundance of O_2_ in an induction furnace prepacked with CuO and platinum catalyst at 900 °C to convert the samples to their combustion products. The combustion products are passed through copper and tungsten catalysts to remove excess O_2_ and to convert nitrous oxides to N_2_ and a moisture trap prepacked with Sicapent to remove moisture interference. An optimized isocratic column oven temperature program starting at 60°C, holding and maintaining it for six minutes at 60°C, is used to elute both the resultant N_2_ and CO_2_ gases with approximately six minutes run per sample. A constant carrier gas (He) flow rate of 1000 ml/minute is maintained throughout the temperature program and N_2_ and CO_2_ are detected by a thermal conductivity detector (TCD).

(2) $$\begin{array}{c} \frac{b-a\;x\;0.1\;x\;v\;x\;100}{1000\;x\;w\;x\;al}\text{,} \end{array}$$

where *a* = volume of the titre HCl for the blank, *b* = volume of titre HCl for the sample taken, and al = aliquot of the solution taken for analysis.

For organic carbon, 0.3 g of the dried sample was digested using 7.5 ml sulphuric acid and 5 ml aqueous potassium dichromate (K_2_CR_2_O_7_) mixture. Unused K_2_CR_2_O_7_ was titrated against ferrous ammonium sulphate to the endpoint denoted by a color change from green to brownish. The concentration of organic carbon was calculated according to [25] in the following equation:

(3) $$\begin{array}{c} carbon=\frac{\left(0.003\;x\;0.2\;\left(Vb-Vs\right)\;x\;100\right)}{w}\text{,} \end{array}$$

where Vb = volume in ml of 0.2 M ferrous ammonium sulphate used to titrate reagent blank solutions; Vs = volume in ml of 0.2 M ferrous ammonium sulphate used to titrate sample solution, and *w* = 12/4000 mili-equivalent weight of C in grams.

### 2.6. Fluxes of CH_4,_ CO_2_, and N_2_O Emitted from Organic and Inorganic Fertilized Nile Tilapia Ponds

The gas samples were subjected to chromatography Model 8610C, SRI (2.74 m Hayasep-D column) at the Mazingira Centre, ILRI. The column was fitted with a 63 Ni-electron capture detector for N_2_O and a flame ionization detector for CH_4_, and CO_2_ was passed through a methanizer. The courier gas used was N_2_ with a flow rate of 20 ml·min^−1^ for the three gases (CH_4,_ CO_2_, and N_2_O). The concentrations of the gases in the glass vials were calculated from the peak areas of standard gases with known concentrations.

Headspace gas concentration changes over time were plotted to produce a slope, and the slope was used to calculate flux [23] in the following equation:

(4) $$\begin{array}{c} f\left(mg\;m^{-2}h{\;}^{-1}\right)=Ct\times \left(\frac{M}{Vm}\right)\times \left(\frac{Vch}{Ach}\right)\times \left(\frac{273.15}{Vm273.1+t}\right)\times P\times 60\text{,} \end{array}$$

where Ct = slope derived from the linear regression (ppmmin−1) for CH_4_ and CO_2_ and (ppb min−1) for N_2_O-N, M = molar weight (g mol−1) (C = 12 for CH_4_ and CO_2_, and N = 28 for N_2_O), Vm = molar gas volume (m1mol−1) (22.41), Vch = volume of the chamber headspace (30 liters), Ach = area of the gas chamber, t = chamber temperature (°C), P = pressure atthe time of sampling (atm), and 60 = conversion factor of minutes to hour.

### 2.7. Data Analysis

All data were analyzed using SPSS version 26 and considered significant at *P*=0.05. Descriptive statistics (mean and standard deviation) was used to describe basic data. One-way ANOVA compared the treatment means for parametric data (physicochemical parameters), while Kruskal–Wallis test compared the nonparametric data (fluxes of the gases). Turkey's test was used to separate the mean differences. Pearson correlation was used to analyze the relationship between greenhouse gas emissions and physicochemical parameters, while the relationship between CH_4_, CO_2_, and N_2_O with time was shown by regression analysis.

## 3. Results

### 3.1. Physicochemical Characteristics of Pond Water from Organic and Inorganic Fertilized Nile Tilapia Ponds

The lowest temperature of 22.22°C was recorded in OF, while the highest was 30.4°C in IF. The temperature of the UF (26.82 ± 1.96)°C was higher than all the other treatments. Statistically, there was no significant difference in temperature among the treatments (*P*=0.899).

The lowest pH of 4.8 was recorded in IF, while the highest (8.9) was recorded in OF. pH in UF (7.70 ± 0.66) remained highest and pH in IF (7.46 ± 1.26) lowest; however, there was no significant difference in pH based on the fertilization (*P*=0.697).

The lowest dissolved oxygen of 2.20°mgl^−1^ was recorded in IF, while the highest (7.49°mgl^−1^) was recorded in OF. The highest overall dissolved oxygen (4.79 ± 1.52°mgl^−1^) was recorded in IF treatments and lowest in UF (4.23 ± 1.36°mgl^−1^). There was no significant difference in dissolved oxygen among the treatments (*P*=0.529).

The lowest conductivity of 46°*µ*s/cm was recorded in OF, while the highest (157°*µ*s/cm) was recorded in UF. The average highest conductivity (90.39 ± 26.47°*µ*s/cm) was reported in IF and lowest in OF (78.90 ± 16.70°*µ*s/cm). There was no significant difference in conductivity based on the fertilization (*P*=0.390) (Table 2).

The lowest CN ratio of 10.06 and highest (29.33) were recorded in OF. The highest average CN ratio of 19.22 ± 6.04 was recorded in IF ponds. There was a significant difference in the CN ratio based on fertilization (*P*=0.025), with UF being significantly lower than IF and OF (Table 2).

Temperature had a positive correlation with CH_4,_ CO_2_, and N_2_O (*r* = 0.33, *P*=0.177; *r* = 0.7, *P*=0.001; and *r* = 0.369, *P*=0.132), respectively. Dissolved oxygen had a slightly negative correlation with CH_4_ (*r* = −0.122, *P*=0.628) and N_2_O (*r* = −0.208, *P*=0.408) and almost no correlation with CO_2_ (*r* = 0.047, *P*=0.852). pH had almost no correlation with CH_4_, and CO_2_ (*r* = 0.037, *P*=0.883; *r* = −0.01, *P*=0.968, respectively) and a slight positive correlation with N_2_O (*r* = 0.142, *P*=0.575). A slight positive correlation was exhibited between conductivity and CH_4_, CO_2_, and N_2_O (*r* = 0.144, *P*=0.568; *r* = 0.212, *P*=0.398; *r* = 0.126, *P*=0.618), respectively. CN ratio positively correlated with CH_4_, CO_2_, and N_2_O (*r* = 0.672, *P*=0.002; *r* = 0.877, *P*=0.0001; *r* = 0.625, *P*=0.006), respectively (Table 3).

### 3.2. Fluxes of CH_4_, CO_2_, and N_2_O Emitted from Organic and Inorganic Fertilized Nile Tilapia Ponds

The lowest CH_4_ flux of 0.001 mg·C·m^−2^h ^−1^ was recorded in UF, while the highest (0.375 mg·C·m^−2^h^−1^) was recorded in OF. The highest mean fluxes of CH_4_ (0.059 ± 0.094 mg·C·m^−2^h ^−1^) were recorded in OF ponds and the lowest (0.010 ± 0.012 mg·m^−2^h^−1^) in UF ponds. There were significant differences in CH_4_ fluxes based on fertilization (*P*=0.005), with UF recording lower flux (Table 4).

The lowest CO_2_ flux (−0.180 mg CO_2_ m^−2^h^−1^) was recorded in UF, while the highest (1.746 mg CO_2_ m^−2^h^−1^) recorded in OF. The highest mean fluxes of CO_2_ (0.334 ± 0.154 mg CO_2_ m^−2^h^−1^) were recorded in OF, while the lowest (0.215 ± 0.407 mg CO_2_ m^−2^h^−1^) recorded in UF. Statistically, there was no significant difference in CO_2_ fluxes based on fertilization (*P*=0.334) (Table 4).

The lowest N_2_O fluxes of −0.628 *µ*g·N·m^−2^h^−1^ were recorded in UF, while the highest (1.383 *µ*g·N·m^−2^h ^−1^) in OF. The highest mean fluxes of N_2_O (0.093 ± 0.324 *µ*g·N·m^−2^h^−1^) were recorded in OF, while the lowest mean fluxes (−0.003 ± 0.175 *µ*g·N·m^−2^h^−1^) were recorded in UF. There were no significant differences in fluxes of N_2_O based on fertilization (*P*=0.696) (Table 4).

**Figure 5:**
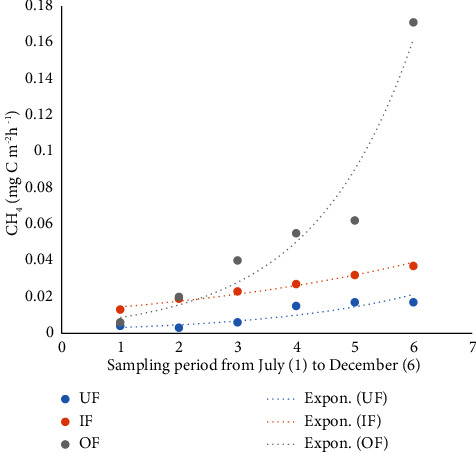
Graph showing fluxes of CH_4_ from unfertilized (UF), inorganic fertilized (IF), and organic fertilized (OF) Nile tilapia ponds.

**Figure 6:**
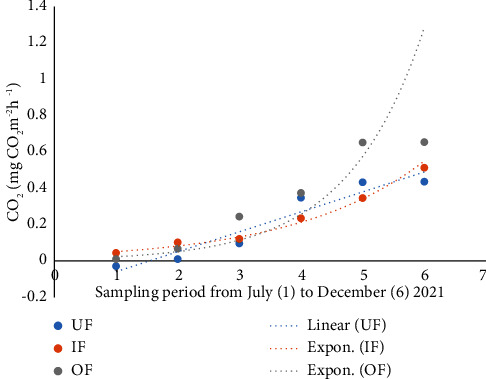
Graph showing fluxes of CO_2_ from unfertilized (UF), inorganic fertilized (IF), and organic fertilized (OF) Nile tilapia ponds.

**Figure 7:**
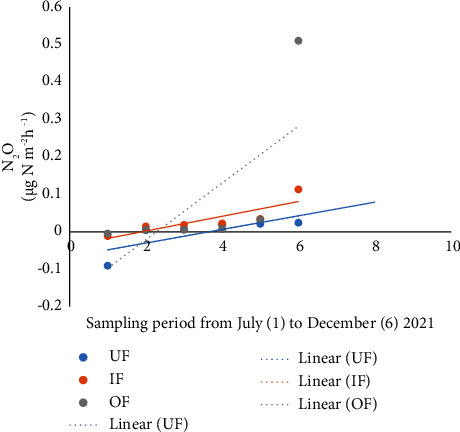
Graph showing fluxes of N_2_O from unfertilized (UF), inorganic fertilized (IF), and organic fertilized (OF) Nile tilapia ponds.

There was a steady increase in CH_4_ (Figure 5), CO_2_ (Figure 6), and N_2_O (Figure 7) with time. When the growth of GHG data was subjected to CH_4_, CO_2,_ and N_2_O, time regression, the greatest increase in emissions of CH_4,_ CO_2,_ and N_2_O with time was observed in the OF ponds.

## 4. Discussion

The study was set to investigate the effect of fish pond fertilization on CH_4_, CO_2,_ and N_2_O emissions. Our study revealed that on average, the fish ponds emitted CH_4_, CO_2,_ and N_2_O, except UF which did not emit N_2_O. Organic fertilization of fish ponds appears to result in high GHG fluxes, because of the high nutrient load exhibited by CN. High organic matter decomposes, thereby utilizing oxygen and creating anaerobic conditions at the pond bottom [26], enabling methanogens to consume the matter, and releases CH_4_. At the same time, on the upper aerobic layers of the pond, methanotrophic bacteria oxidize CH_4_ to produce CO_2._ In this study, there is a strong positive correlation between CH_4_ and CO_2_ (*r* = 0.688; *P*=0.002), pointing to the possible fact of methanotrophic bacterial activity in the ponds. Furthermore, the availability of more nutrients supplies extra substrates for N_2_O production [27], which is also important in primary production. The UF, which was typically limited in substrates, may have influenced the average N_2_O production, as these ponds acted as sinks and not as emitters of N_2_O.

Nevertheless, the study recorded lower daily fluxes for CH_4_ m^−2^d^−1^ than those recorded in tropical and subtropical surface water bodies in previous studies [28–32]. It is possible that this difference could be attributed to the quality of organic loads in the water bodies [33]. These fertilizers are introduced in the fish pond water, which forms the immediate environment for fish and is very important as it forms the basis of fish metabolism [12]. The breakdown of the different organic loads consumes oxygen, thereby creating anoxic environments that favor CH_4_ emissions in the pond bottom as CO_2_ is released on the upper aerobic pond layers when CH_4_ is oxidized [26].

Water quality plays an important role in GHG emissions. The increase in temperature has been shown to increase CH_4_ emissions resulting from enhanced methanogen activity [26, 34]. Additionally, increase in temperature reduces dissolved oxygen and eventually increases CH_4_ emissions [35].

CO_2_ fluxes significantly correlated positively with temperature, an indication that the mineralization of organic matter remains a key factor in determining the release of CO_2_ as heterotrophy was higher than autotrophy [36]. The negative correlation shown between pH and CO_2_ is attributed to the fact that as CO_2_ accumulates in water, it leads to a fall in pH levels [37]. According to [32], the acidic water favors a high flux of pond water to the atmosphere.

The addition of fertilizer led to an increase in NO_2._ This could be as a result of the supply of more nitrogen substrate for decomposers leading to higher NO_2_ emissions. However, when the fluxes of the three GHGs were compared, NO_2_ had the lowest fluxes. This could be explained by the fact that there exist strict anoxic conditions in the pond, which enhance denitrification, leading to the end product of nitrogen gas instead of NO_2_ [38]. N_2_O fluxes positively correlated with temperature and negatively correlated with dissolved oxygen. Higher water temperature enhances the denitrification process through oxygen demanding metabolic pathways [39, 40].

The CN ratio was noted to be a driver and determinant of GHG emissions owing to its strong significant positive correlation with CH_4_, CO_2,_ and N_2_O. The CN ratio influences the mineralization and immobilization processes.

It can be noted that the created wetlands (artificial wetland in our case) provide food for fish through aquaculture as explained in [41]. This creation of artificial wetlands is a land use change, which leads to the loss of the soil's ability to sequestrate carbon, leading to more emissions. However, the emissions in our study are much lower than the emissions from crop fields. The mean fluxes reported by [42] for most of the East African soils were 1.2–10.1 kg·C·ha, 3.5–15.9 g·C·m^−2^h^−1^, and 2–62 *µ*g·N·m^−2^h^−1^ for CH_4_, CO_2_, and N_2_O, respectively. This shows that fish ponds have not been used long enough to accumulate a higher organic load compared to agricultural soils, which have been used for long periods. This is also supported by observations made by Singh et al. [43], who did a comparison of natural and artificial ponds in India and noted lower CH_4_ fluxes in artificial ponds. This was because the artificial ponds had lower nutrient concentrations and less sediment. Wetlands accumulate organic carbon in the soil due to anaerobic conditions leading to slower decomposition compared to crop lands [44]. During the cultivation of agricultural land, the soil organic carbon is subjected to more favorable conditions for decomposition (aerobic), hence enhancing more emissions [45].

Though semi-intensive Nile tilapia production system using fertilization increases GHG emissions, it is important to note that the emissions produced in the production of other related animal protein foods are much higher compared to Nile tilapia which is 1.58 kg CO_2_ [46]. 45.54 and 2.4 kg CO_2_ were emitted in the production of 1 kg of mutton and milk, respectively [46], while 2.179 was emitted to produce 1 kg eggs [47]. These observations agree with [15] that aquaculture could offer a good alternative for achieving food security under the changing climate.

## 5. Conclusions and Recommendations

The study assessed methane (CH_4_), carbon dioxide (CO_2_), and nitrous oxide (N_2_O) emissions and physicochemical parameters arising from Nile tilapia fish ponds fertilized with organic and inorganic fertilizers. The three fertilization treatments significantly affected the CN ratio and CH_4_ emissions. However, fertilization treatments had no significant effect on CO_2_ and N_2_O emissions, temperature, pH, dissolved oxygen, and conductivity. Even though the fertilization treatments were not significant for CO_2_ and N_2_O, there were much higher fluxes of CO_2_ and N_2_O in IF and OF than in UF. The authors conclude that fish pond fertilization increases emissions of greenhouse gases. However, it is good to note that Nile tilapia production produces much less emissions compared to other animal proteins and remains a viable way to offer food security, nutrition, and income to the smallholder fish farmers in the changing environments. Since a large number of farmers depend on organic pond manuring for improvement in production, there is a need for developing technologies of fish pond manure processing and management to ensure fish production units in the region.

## Figures and Tables

**Table 1:** Proximate component of fish feed.

Component	% content
Crude proteins	42.50
Fats	7.64
Fiber	13.13
Moisture	8.52
Ash	18.94

**Table 2:** Mean physicochemical parameters from unfertilized, organic fertilized, and inorganic fertilized Nile tilapia fish ponds over 6 months (with range in parenthesis).

Parameters	UF mean ± SD	IF mean ± SD	OF mean ± SD	*P* value	NEMA STDS
*Physical*					
Temp. (°C)	26.82 ± 1.96^a^ (23.39–29.58)	26.50 ± 2.04^a^ (22.69–30.40)	26.60 ± 2.30^a^ (22.22–30.07)	0.899	20–35
pH	7.70 ± 0.66^a^ (6.50–8.78)	7.46 ± 1.26^a^ (4.80–8.78)	7.67 ± 0.73^a^ (6.00–8.90)	0.697	6.5–8.5

*Chemical*					
DO (mgl^−1^)	4.23 ± 1.36^a^ (2.40–6.88)	4.79 ± 1.52^a^ (2.20–6.60)	4.47 ± 1.57^a^ (2.60–7.48)	0.529	>4
Cond. (*µ*s/cm)	85.28 ± 29.74^a^ (48.00–157.00)	90.39 ± 26.47^a^ (49.00–130.00)	78.90 ± 16.7^a^ (46.00–101.00)	0.390	—
CN ratio	14.45 ± 3.94^a^ (11.67–29.33)	19.22 ± 6.04^ab^ (10.67–28.50)	18.57 ± 6.00^b^ (10.06–29.33)	0.025	—

Means in a row with the same superscripts indicate no significant difference at 5%.

**Table 3:** Correlation matrix of Nile tilapia weight, emissions, and physicochemical parameters.

	Temp.	DO	pH	Cond.	CN	CH_4_	CO_2_	N_2_O
Temp.	1.0000							

DO	−0.134	1.0000						
	0.597							

pH	0.227	−0.321	1.0000					
	0.365	0.194						

Cond.	0.275	0.332	−0.290	1.0000				
	0.269	0.179	0.243					

CN	0.629^*∗∗*^	0.164	0.031	0.307	1.0000			
	0.005	0.516	0.904	0.215				

CH_4_	0.33	−0.122	0.037	0.144	0.672^*∗∗*^	1.0000		
	0.177	0.628	0.883	0.568	0.002			

CO_2_	0.700^*∗∗*^	0.047	−0.01	0.212	0.877^*∗∗*^	0.688^*∗∗*^	1.0000	
	0.001	0.852	0.968	0.398	0.0001	0.002		

N_2_O	0.369	−0.208	0.142	0.126	0.625^*∗∗*^	0.919^*∗∗*^	0.603^*∗∗*^	1.0000
	0.132	0.408	0.575	0.618	0.006	0.0001	0.008	

^*∗*^Correlation is significant at 0.05 level (2-tailed); ^*∗∗*^correlation is significant at 0.01 level (2-tailed).

**Table 4:** Mean fluxes of CH_4_, CO_2,_ and N_2_O from unfertilized (UF), inorganic fertilized (IF), and organic fertilized (OF) Nile tilapia ponds (with range in parenthesis) over a 6-month period.

Parameters	UF mean ± SD	IF mean ± SD	OF mean ± SD	*P* value	NEMA STDS
CH_4_ (mg·C·m^−2^h^−1^)	0.010 ± 0.012^a^ (0.001–0.043)	0.025 ± 0.020^b^ (0.005–0.068)	0.059 ± 0.094^b^ (0.001–0.375)	0.005	—
CO_2_ (mg·CO_2_·m^−2^h^−1^)	0.216 ± 0.407^a^ (−0.180–1.400)	0.227 ± 0.278^a^ (−0.020–1.101)	0.334 ± 0.454^a^ (−0.049–1.746)	0.344	4 mg CO_2_·m^−3^h^−1^
N_2_O (*µ*g·N·m^−2^h^−1^)	−0.003 ± 0.175^a^ (−0.628–0.326)	0.032 ± 0.056^a^ (−0.049–0.187)	0.093 ± 0.324^a^ (−0.022–1.384)	0.696	6.25 *µ*g·m^−3^h^−1^

Means in a row with the same superscripts indicate no significant difference at 5%.

## Data Availability

The data used to support the findings of this study are available from the corresponding author upon request.
